# Clinical Course and Outcome of Non-Immune Fetal Hydrops in Singleton Pregnancies

**DOI:** 10.3390/jcm11030702

**Published:** 2022-01-28

**Authors:** Theresa Reischer, Bernadette Muth, Anja Catic, Cécile Monod, Tina Linder, Christian Göbl, Gülen Yerlikaya-Schatten

**Affiliations:** 1 Department of Obstetrics and Gynecology, Medical University of Vienna, 1090 Vienna, Austria; theresa.reischer@meduniwien.ac.at (T.R.); muth.berni@gmail.com (B.M.); anja.catic@meduniwien.ac.at (A.C.); tina.linder@meduniwien.ac.at (T.L.); christian.goebl@meduniwien.ac.at (C.G.); 2 Department of Obstetrics and Gynecology, University Hospital Basel, 4031 Basel, Switzerland; Cecile.monod@meduniwien.ac.at

**Keywords:** non-immune fetal hydrops, prenatal diagnosis, outcome

## Abstract

Nonimmune fetal hydrops is a condition defined by abnormal fluid accumulation in two or more body compartments. The aim is to evaluate factors associated with adverse outcome in diagnosed fetal hydrops and to investigate the aspects for the decision making in the case of termination of pregnancy. Therefore, a retrospective data analysis of pregnancies complicated by non-immune hydrops fetalis between 2004 and 2018 was performed in a single tertiary referral center. Of 361 pregnancies with diagnosed fetal hydrops, in 183 cases (50.7%), the parents decided to terminate the pregnancy. A strong relationship between etiology and termination of pregnancy was demonstrated, whereas the highest rates of termination of pregnancy were found if a chromosomal aberration was diagnosed. Of the remaining 178 cases, 51 cases (28.7%) had a miscarriage, 33 cases (18.5%) had an intrauterine fetal death, and 94 cases (52.8%) were live born, whereas 26 (27.7%) of these offspring died within the first week of life. The risk of an adverse outcome increased with lower gestational age at diagnosis (*p* < 0.001). A nuchal translucency thickness greater than 2.5 mm was associated with an adverse outcome (*p* < 0.01). Furthermore, pregnancies with adverse outcome had significantly more affected compartments (median: 3; IQR 2), compared with live born cases (median: 2; IQR 1; *p* < 0.01). In conclusion, adverse outcome in pregnancies with fetal hydrops was associated with a lower gestational age at diagnosis, nuchal translucency greater than 2.5 mm and a higher count of affected compartments. These results confirm that a precise clinical workup to identify the underlying etiology of non-immune fetal hydrops is essential for a better prognostic assessment and accurate counselling of parents.

## 1. Introduction

Hydrops fetalis is a condition characterized by abnormal fluid accumulation within two or more compartments and body cavities of the fetus [1]. It is classified as either immune hydrops fetalis, due to rhesus alloimmunization, or non-immune hydrops fetalis (NIHF), whereas NIHF is the cause in more than 85% of fetuses [2,3].

NIHF has a causal relationship with hypoproteinemia and intrauterine heart failure. In connection with hypoproteinemia, it is assumed that alpha-fetoprotein (AFP) takes over the function of albumin in the fetal organism and is therefore an important factor in fetal plasma osmolarity [4].

Furthermore, hydrops fetalis has its origin in a variety of chromosomal abnormalities, skeletal dysplasia, and structural abnormalities that interfere with fetoplacental circulation [5]. Despite extensive research, the etiology of NIHF may remain unknown in up to 25% of cases [6,7], although a growing number of conditions have been identified as the cause for hydrops fetalis.

Despite better understanding of this condition and improvement of diagnosis and possible treatments of NIHF, the mortality remains extremely high [8,9].

Hydrops fetalis can be found as early as in the first trimester of pregnancy. In this context, hydrops fetalis is often associated with increased nuchal translucency (NT), with or without so-called cystic hygroma colli. It is assumed that screening for aneuploidies has likely reduced the relative contribution of chromosomal abnormalities to NIFH over the years [7]. In addition, advances in prenatal diagnosis may have led to an improvement in identifying the cause of hydrops fetalis. Furthermore, new genes are identified continually to be associated with NIFH [10].

The aim of the study was to investigate the clinical course and pregnancy outcome of all fetuses diagnosed with NIHF Furthermore, (i) to investigate pregnancy outcome for fetuses with increased nuchal translucency (NT) ≥ 2.5mm and hydrops in particular, (ii) to determine factors associated with the decision on termination of pregnancy if fetal hydrops was diagnosed, and (iii) to investigate factors associated with an adverse outcome in fetuses with hydrops if parents decided to continue the pregnancy.

## 2. Materials and Methods

A retrospective cohort study with all cases of NIHF between 2004 and 2018 at a tertiary referral center was performed. An invasive procedure for genetic analysis including karyotype, chromosomal microarray analysis and, in selected cases, whole-exome sequencing, was offered to every patient and all women had a detailed scan, including Doppler measurement of the MCA after 19 weeks (wks), which was performed by a fetal medicine specialist. All patients were followed up until delivery. Pregnancy outcome was recorded and defined as miscarriage (MC) before 20 wks, termination of pregnancy (TOP), intrauterine fetal death (IUFD) after 20 wks, and perinatal death (death within the first week of live) or live birth. For some of the analysis MC, IUFD, and perinatal death were pooled as adverse outcomes. Subsequently, the cause of NIHF was classified into the following categories: chromosomal aberration (including aneuploidies, duplication, and deletions); cardiovascular (including arrythmia, complex congenital heart defect, cardiomegaly), syndromic (for example, Noonan syndrome), infectious (parvovirus), thoracic (including congenital diaphragmatic hernia, congenital cystic adenomatoid malformation), hematological, fetal akinesia, skeletal dysplasia, urogenital, gastrointestinal or unknown. NT in the first trimester scan between 11 and 14 weeks of gestation was reviewed. Between 18 and 22 wks, all cases that were not terminated or had a miscarriage before had a mid-trimester anomaly scan, according to the practice guideline of the International Society of Ultrasound in Obstetrics and Gynecology (ISUOG) [11]. Cases of hydrops in monochorionic twins due to twin-to-twin transfusion syndrome and hydrops due to rhesus alloimmunization were excluded.

For statistical analysis, metric variables were presented as the mean ± SD or median and IQR. Metric variables were compared using Student’s *t*-test in the case of a standard distribution (comparison of the mean NT thickness) and Mann–Whitney U-test in the case of variables not following a standard distribution (comparison of affected compartments, gestational age at diagnosis). We used a chi-square test to compare binary and categorical variables. A two-sided *p*-value of <0.05 was set for statistical significance. Binary logistic regression was used to assess the association between adverse outcome, gestational age at diagnosis, affected body compartments, and nuchal translucency. A multivariate logistic regression model was stepwise fitted to test factors associated with adverse outcomes. Statistical analyses were performed using IBM SPSS Static Version 23.

## 3. Results

In this single tertiary prenatal center with an average of 2600 live births per year, we evaluated 361 pregnancies complicated by excessive fluid accumulation over a period of 15 years (total number of live births 38,994). The overall rate of IUFD in this period of time was 1.9%; the rate of perinatal death was 1% (death within the first week of life), and 11% of live born had prenatally diagnosed congenital malformations.

The average maternal age of women with a pregnancy diagnosed with NIFH was 31.5 years (SD ± 6.3), ranging from 17 to 47 years. The median gestational age of clinical suspicion of NIHF was 14.27 (IQR 7.93) wks, ranging from 8.9 to 36.7 wks, influenced by the etiology and the time of referral to our hospital. The median gestational age at diagnosis did not change throughout the years of the investigation. Overall, 51% of women decided to continue the pregnancy, and 49% chose the termination of pregnancy.

### 3.1. Etiology

Chromosomal aberration was identified as the most common cause of NIFH in 42.9% of cases. Most frequently, karyotyping revealed a Turner Syndrome (45,X) in 57 cases, followed by Trisomy 18 in 42 cases, and Trisomy 21 in 40 cases. In 32% (107) of cases, the etiology remained unknown, whereas in 57 of these cases (53.2%), a chromosomal analysis was not available as the parents declined genetic testing. After chromosomal aberration, abnormalities in the cardiovascular system were a major contributor to fetal hydrops. Cardiovascular causes of NIHF, including mainly congenital heart defects, but also arrythmias and cardiomyopathies, were identified in 45 cases (12.5%). Other underlying etiologies affected fewer than 5% of cases and included infectious, hematological, urogenital, gastrointestinal, and thoracic issues as well as skeletal dysplasia, and fetal akinesia syndrome (detailed results are listed in Table 1).

The time of diagnosis differed significantly according to the etiology. For example, the median gestational age (GA) at diagnosis of fetuses with aneuploidies or other chromosomal aberration was 13.3 (IQR 2.1) wks; affected fetuses with a cardiovascular cause were diagnosed at a median GA of 20.1 (IQR 14.0) wks (*p* < 0.001).

### 3.2. Affected Fetal Body Cavity

The most commonly observed compartment affected by abnormal fluid accumulation was the skin, recorded in 85% of fetuses. Cervical hygroma was present in 70%, whereas ascites was present in 43%. Altogether, half of the cases (52.1%) had excessive fluid in two compartments, 31.9% in three, 14.4% in four compartments, and in six cases (1.7%), five compartments were affected.

Cervical hygroma was associated with chromosomal aberration (OR 11.049, 95%CI 5.503–22.185, *p* < 0.001), particularly monosomy X, trisomy 18, and trisomy 21. 68.7% of fetuses with cervical hygroma had an abnormal karyogram. In contrast, ascites and pericardial effusion were predominantly observed in euploid fetuses (66%, 85%). There was no significant difference between chromosomal findings in cases with and without hydrothorax.

### 3.3. Outcome

Overall, 183 (50.7%) patients opted for termination of pregnancy. These rates of ToP did not change throughout the years. The majority of these fetuses had an elevated nuchal translucency or other early ultrasound anomalies.Therefore, 177 (96.7%) patients underwent drug-induced termination of pregnancy at an average gestational age of 15.5 wks. Only six cases (3.3% of ToP) had feticide. Five of these cases were diagnosed later in pregnancy, due to a missing first trimester screening or a normal result after the first trimester screening (one case with congenital diaphragmatic hernia, one case with Mucopolysaccharidosis type 7, two cases with complex cardiac malformation, and one case with deleterious chromosome 21 and 13). One feticide was performed because of a Turner syndrome that had been diagnosed in the first trimester, but failed three attempts of drug-induced ToP.

It is worth noting that the decision on termination of pregnancy was strongly related to the underlying etiology. The leading cause of ToP was a diagnosed chromosomal aberration. This occurred in 62.3%, in comparison to 3.8% of ToP, due to NIFH with cardiovascular etiology. Besides that, the association between wider NT thickness and ToP was statistically significant (OR 0.107, 95%CI 0.045–0.251 *p* < 0.001, Table S2).

Nevertheless, 178 patients decided to continue the pregnancy. In the course of these pregnancies, 51 (28.7%) had a miscarriage and 33 (18.5%) had an intrauterine fetal death after 20 wks. In total, 94 fetuses (52.8%) were live born, whereas 26 (27.7%) of these offspring died within the first week of life.

#### Nuchal Translucency

In a subgroup of 264 pregnancies, data of the first trimester screening, including NT, were available. Of the remaining 97 cases, 41 (11%) declined first trimester screening, and for 56 cases (15.5%), data were not available due to transferal of the patient in a progressed week of pregnancy. The mean NT thickness in the overall cohort was 7.2 mm (ranging from 0.8 to 23.9 mm). A comparison of the mean NT thickness showed a mean NT thickness of 8.2 mm (SD ± 3.8) in cases of ToP and a significantly lower average NT level of 5.6 (SD ± 3.34) mm in the continued pregnancies (OR 0.805, 95%CI 0.741–0.874, *p* < 0.001).

To evaluate the association of NT values with adverse outcome, fetuses were divided in two groups (NT greater than 2.5 mm and less than 2.5 mm). The group with NT values greater than 2.5 mm had a higher risk for adverse fetal outcome (OR 3.31, 95%CI 1.4−7.7 *p* < 0.01). Furthermore, cases were grouped into five NT categories (<2.5, 2.5−4, 4−6, 6−8, >8 mm) to evaluate an association between the rate of adverse outcome and increasing NT thickness. As shown in Table 2, all four groups with NT values greater than 2.5 mm showed comparable high rates of adverse outcome.

The comparison of the median NT thickness between live births and cases with adverse outcome did not reach statistical significance (5.04 vs. 5.79). Pregnancies resulting in miscarriage before 20 wks showed substantially higher NT thickness during the first trimester ultrasound scan with an average thickness of 7.1 mm compared to 4.4 mm in live births. Pregnancies resulting in intrauterine death after 20 wks did not show majorly higher NT thickness compared to live-born cases.

### 3.4. Gestational Age (GA) at Diagnosis

The overall mean GA at diagnosis was 17.3 wks (median 14.3, IQR 7.9). Unsurprisingly, we observed a lower median GA of 13.4 (IQR 2.6) wks in case of ToP as compared to GA at diagnosis of 19.7 (IQR 13.6) wks in continued pregnancies.

The risk of an adverse outcome increased with lower gestational age at diagnosis (OR: 0.86, 95%CI 0.82–0.90, *p* < 0.001). Live-born cases were diagnosed significantly later, with a median GA at diagnosis of 20.6 wks (IQR 10.6), compared to cases with IUFD with a median GA of 20.3 (IQR 6.0) wks (*p* < 0.01) and compared to cases ending in a miscarriage with a median GA of 12.8 (IQR 1.8) wks (*p* < 0.001).

### 3.5. Prognosis

The prognosis of fetuses was strongly associated with the diagnosed etiology of NIHF: Pregnancies with chromosomal aberrations showed the highest rates of miscarriages. All fetuses diagnosed with fetal akinesia syndrome and hydropic features died intrauterine or shortly after birth. Fetuses with NIHF due to thoracic causes such as congenital diaphragmatic hernia (CDH) and congenital cystic adenomatoid malformation (CCAM) showed a comparable good outcome, with a high rate of live births (Table 3).

Furthermore, cases with an adverse outcome had significantly more affected compartments with a median of 3 (IQR 2) compared to live-born cases with a median of 2 (IQR 1) (*p* < 0.01). However, there was no notable difference in the number of affected compartments between miscarriage and live births, but cases with intrauterine fetal death and cases that died perinatally had significantly more affected compartments than live-born cases.

Additionally, adverse outcome was significantly associated with cystic hygroma (OR 4.933, 95%CI 2.559−9.510, *p* < 0.001) and skin edema (OR 9.429. CI95% 4.214−21.098, *p* < 0.001). Other affected compartments such as pericardial effusion, ascites, and hydrothorax did not have an effect on the rate of adverse outcome as a single factor (Table S1).

#### Associated Malformations

Overall, 43.9% of fetuses showed associated structural malformation. The most common associated malformations were congenital heart defect in 52 cases (14.1%). Moreover, a complex malformation syndrome, affecting more than two organ systems, was present in 35 cases (9.7%) overall, including 17 cases of the ToP group and 18 cases where the parents decided to continue the pregnancy.

Pregnancies ending in live birth had more often associated malformations in 41 cases (60.3%) than IUFDs in 13 (39.4%) or miscarriages in 17 cases (33.3%). However, the highest rates of associated malformations in 18 cases (69.2%) were observed in the group of perinatal deaths. Although the presence of one associated malformation was not associated with adverse outcome, complex malformation syndromes showed a significant association with adverse outcome(OR 2.370 95%CI 1.118–5.026, *p* < 0.05). Only two of 18 cases (11%) with multiple malformations were live-born.

In summary, ToP was associated with lower GA at diagnosis, the type etiology, and higher NT thickness. Lower GA at diagnosis, NT > 2.5 mm, as well as the count and the type of affected compartments were identified as factors associated with adverse outcome. A detailed summary of these findings is shown in Table S2.

With a multivariate logistic regression model, we evaluated the association between several factors associated with adverse outcome (miscarriage, IUFD, and perinatal death) in the 178 cases where the parents decided to continue the pregnancy. We identified GA at diagnosis and number of affected body compartments as independent risk factors for adverse outcome (Table 4).

## 4. Discussion

We evaluated the clinical course of 361 pregnancies with the diagnosis of NIFH. The most common etiology identified prenatally was an abnormal karyotype in 42.9%, which is notably higher than the rate in the systematic review of Bellini of 12.5% [3]. This might be explained by the fact that many cases with chromosomal aberration would also fit into another category because of known associations of structural malformations with chromosomal aberrations, for example, heart defects. In this investigation, all cases with an abnormal karyotype, independent of the associated malformation, were included in the category of chromosomal aberration. While chromosomal aberration was identified as the most common cause of NIFH, the reason for NIFH in 32% (107) of cases remained unknown, whereas in 57 cases (53.2%), a chromosomal analysis was not available as the parents declined genetic testing. By excluding these cases, the etiology of NIFH could be identified in 83.6% (254/304), resulting in 16.4% of NIFH with an idiopathic etiology, which is slightly lower than but comparable to the published literature value of 19.8% [2].

The rate of ToP in our cohort with 50.8% (183 cases) is consistent with previously published literature (44.3%) [12]. The decision on ToP was associated with etiology, elevated NT thickness, and GA at diagnosis. In our study population, the highest rate of ToP with 72.5% was observed if an underlying genetic cause was diagnosed prenatally. While literature concerning ToP in fetal hydrops is sparse, many investigations have already confirmed high rates of ToP in the case of prenatally detected chromosomal aberration, especially if trisomy 21 was diagnosed [13,14,15]. The association of early diagnosis and ToP is consistent with the results from Sileo et al. [12] and relatable due to the expected worse outcome and emotional factors later in pregnancy such as the sense of fetal movement.

However, the fact that earlier diagnosis was associated with adverse outcome was also demonstrated in this study and represents a central aspect in counselling affected parents. Moreover, in this population of NIFH, NT thickness greater than 2.5 mm had a remarkable association with adverse pregnancy outcome. Noteworthy, the rate of adverse fetal outcome did not further increase with increasing NT thickness greater than 2.5 mm. In contrast, studies investigating NT thickness independent of the presence of NIFH showed decreasing rates of live born in cases of increasing NT thickness [16,17,18]. This might be explained by the fact that highest rates of ToP were observed in the categories with higher NT thickness, which could be a selection bias in our cohort.

Furthermore, we demonstrated that the number of affected body compartments was associated with adverse pregnancy outcome (2 vs. 3). Our results confirm the previously published findings of Kim et al. Comparable with our data, they reported significantly more affected body compartments in cases with adverse outcome (non-survivors) compared to live-born cases. Additionally, they demonstrated a significant increase in perinatal mortality if more than three body compartments were affected, with the limitation of a notably smaller cohort of 43 fetuses [19].

Another point worth mentioning is the relationship of etiology and the fetal outcome. Fetuses with diagnosed aneuploidy and copy number variations showed worse outcome than euploid fetuses with cardiovascular or thoracic cause of NIFH, which could most likely be treated postpartum. If genetic analysis revealed a chromosomal aberration, it was easier for the clinician to make a prognosis about the course and outcome of the pregnancy than if genetic diagnosis revealed a normal result. Interestingly, high rates of associated malformations were found in live births. This might be explained by the fact that associated malformations are more likely to be identified later in pregnancy. Additionally, in this investigation, we did not distinguish between major and minor malformation. Furthermore, the presence of a complex malformation syndrome was strongly associated with adverse outcome.

In this cohort, there was only a small number of cases with complete genetic analysis including whole-exome sequencing (WES). In nine cases, WES helped to identify a monogenetic genetic disease; for example, in two unrelated cases, pathogenetic variants in the *GUSB* gene were identified, leading to the diagnosis of mucopolysaccharidosis type 7. In two other cases, Noonan syndrome was identified by heterozygous variants in the *PTPN11* gene and the *RIT1* gene, respectively. Recent studies already showed the advantage of further genetic follow-up using exome sequencing. These investigations showed diagnostic yields of exome sequencing in fetal hydrops between 29 and 42.1% [20,21,22], suggesting that exome sequencing will play an increasing role in the clinical workup of NIFH.

### Strength and Limitations

This investigation is one of the few studies with a large sample size of 361 cases in one single center. One of the main limitations is the retrospective design with the risk of case selection and the lack of information after the perinatal period. Another limitation is the incomplete genetic workup due to the availability of genetic analysis at different timepoints. The high rate of ToP might be a selection bias, as in severe cases, parents rather opt for ToP. On the other hand, pregnancies were continued in 178 cases, which is still a higher sample size compared with most of the published investigations.

Nevertheless, despite the partially missing genetic testing and the retrospective evaluation, representative statements can still be made about the clinical course and outcome of NIFH due to the high number of cases.

## 5. Conclusions

In conclusion, we demonstrated that GA at diagnosis, etiology, elevated NT as well as count and type of affected compartments were associated with adverse outcome in cases with diagnosed NIFH. Therefore, a precise clinical workup and genetic analysis are essential for accurate counselling of future parents.

## Figures and Tables

**Table 1:** Etiology of NIFH (nonimmune fetal hydrops) and rate of ToP (termination of pregnancy).

Etiology	*N* (%)	TOP N (%)
Aneuploidy	155 (42.9%)	114 (73.1%)
Deletions/Duplication	6 (1.7%)	3 (75.0%)
Syndromic	5 (1.4%)	7 (63.6%)
Cardiovascular	45 (12.5%)	11 (25.0%)
Infectious	6 (1.7%)	3 (50.0%)
Thoracic	9 (2.5%)	1 (11.1%)
Haematological	3 (0.8%)	0.0%
Fetal akinesia	5 (1.4%)	3 (50.0%)
Skeletal dysplasia	9 (2.5%)	8 (88.9%)
Urogenital	5 (1.4%)	0.0%
Gastrointestinal	6 (1.7%)	0.0%
Unknown	107 (29.6%)	33 (32.0%)

**Table 2:** Association between nuchal translucency (NT) thickness and adverse outcome.

NT Thickness (mm)	Live Births	Adverse Outcome
<2.5	11 (39.3%)	17 (60.7%)
2.5−4	1 (9.1%)	10 (90.9%)
4.1−6	3 (15.8%)	16 (84.2%)
6.1−8	7 (24.1%)	22 (75.9%)
>8	4 (21.1%)	15 (78.9%)

**Table 3:** Comparison of outcome in different etiologies of NIFH (nonimmune fetal hydrops).

Etiology	Live Birth	Perinatal Death	Intrauterine Fetal Death (IUFD)	Miscarriage (MC)
Aneuploidy	9 (21.4%)	1(2.4%)	8 (19.0%)	24 (57.1%)
Deletions/Duplication	0.0%	0.0%	1 (100.0%)	0.0%
Syndromic	2 (50.0%)	2 (50.0%)	0.0%	0.0%
Cardiovascular	17 (51.5%)	8 (24.2%)	7 (21.2%)	1 (3.0%)
Infectious	1 (33.3%)	0.0%	2 (66.7%)	0.0%
Thoracic	7 (87.5%)	0.0%	1 (12.5%)	0.0%
Haematological	2 (66.7%)	0.0%	1 (33.3%)	0.0%
Fetal akinesia	0.0%	2 (66.7%)	1 (33.3%)	0.0%
Skeletal dysplasia	1 (100.0%)	0.0%	0.0%	0.0%
Urogenital	2 (50.0%)	1 (25.0%)	0.0%	1 (25.0%)
Gastrointestinal	5 (83.3%)	1 (16.7%)	0.0%	0.0%
Unknown	22 (31.4%)	11 (15.7%)	12 (17.1%)	25 (35.7%)

**Table 4:** Factors associated with adverse outcome. ns: not significant.

Univariate Analysis	OR	95%CI	*p*-Value
Compartments affected	1.66	1.13–2.44	*p* < 0.01
Gestational age at diagnosis	0.86	0.82–0.90	*p* < 0.001
Nuchal translucency > 2.5	3.30	1.42–7.69	*p* < 0.01
Chromosomalaberration(Y/N)	2.49	1.10–5.64	*p* < 0.05
Malformations (Y/N)	1.02	0.82–1.24	ns
**Multivariate analysis**			
Gestational age at diagnosis	0.84	0.80–0.89	*p* < 0.001
Compartments affected	2.22	1.38–3.57	*p* < 0.001

## Data Availability

The data presented in this study are available on request from the corresponding author. The data are not publicly available due to data privacy.

## Supplementary Materials

The following are available online at https://www.mdpi.com/article/10.3390/jcm11030702/s1, Table S1: Association of types of affected compartments with adverse outcome, Table S2: Summarized findings: factors associated with adverse outcome and termination of pregnancy (ToP).
