# Supplementary material for: Clinical Course and Outcome of Non-Immune Fetal Hydrops in Singleton Pregnancies
Source: J Clin Med. 2022 Jan 28;11(3):702. doi: 10.3390/jcm11030702 (PMC8836777; doi:10.3390/jcm11030702)
Supplement: Supplementary file 1 [file jcm-11-00702-s001.zip › jcm-1528079-supplementary.pdf]

Supplementary Materials

Table S1. Association of types of affected compartments with adverse outcome.

**Affected Compartment**		**Live Birth**	**Adverse Outcome**	***p*-Value**
pericardal effussion	not present	48 (34.3%)	92 (65.7%)	
	present	20 (52.6%)	18 (47.4%)	p=0.059
hydrothorax	not present	29 (38.7%)	46 (61.3%)	
	present	39 (37.9%)	64 (62.1%)	p=0.913
skin edema	not present	33 (76.7%)	10 (23.3%)	
	present	35 (25.9%)	100 (74.1%)	p<0.001
ascites	not present	20 (29.4%)	48 (70.6%)	
	present	48 (43.6%)	62 (56.4%)	p=0.058
cervical hygroma	not present	48 (57.1%)	36 (42.9%)	
	present	20 (21.3%)	74 (78.7%)	p<0.001

Table S2. Summarized findings: factors associated with adverse outcome and termination of pregnancy (ToP).

		**Live Birth**	**Adverse Outcome**	***p*-Value**
chromosomal aberrations	not present	59 (42.8%)	79 (57.2%)	
	present	9 (23.1%)	30 (76.9%)	p<0.05
NT thickness	< 2.5 mm	17 (48.6%)	18 (51.4%)	
	>2.5 mm	18 (22.2%)	63 (77.8%)	p<0.01
skin edema	not present	33 (76,7%)	10 (23,3%)	
	present	35 (25,9%)	100 (74,1%)	p<0.001
cervical hygroma	not present	48 (57.1%)	36 (42.9%)	
	present	20 (21.3%)	74 (78.7%)	p<0.001
GA at diagnosis	median (IQR)	25.7 (9.9)	15.1(8.4)	p<0.001
affected compartments	median (IQR)	2 (1)	3 (2)	p<0.01
		**ToP**	**continued pregnancies**	**p-value**
chromosomal aberrations	not present	72 (34.3%)	138 (65.7%)	p<0.001
	present	100 (73.8%)	39 (26.2%)	
NT thickness	< 2.5 mm	7 (16.7%)	35 (83.3%)	
	>2.5 mm	152 (65.2%)	81 (34.8%)	p<0.001
GA at diagnosis	median (IQR)	13.4 (2.6)	19.7 (13.6)	p<0.001
